# Plant Resources as a Factor Altering Emergent Multi-Predator Effects

**DOI:** 10.1371/journal.pone.0138764

**Published:** 2015-09-25

**Authors:** Dionyssia A. Maselou, Dionyssios Ch. Perdikis, Maurice W. Sabelis, Argyro A. Fantinou

**Affiliations:** 1 Laboratory of Ecology & Environmental Sciences, Agricultural University of Athens, Athens, Greece; 2 Laboratory of Agricultural Zoology and Entomology, Agricultural University of Athens, Athens, Greece; 3 Institute for Biodiversity and Ecosystem Dynamics (IBED), University of Amsterdam, Amsterdam, The Netherlands; French National Institute for Agricultural Research (INRA), FRANCE

## Abstract

Multiple predator effects (MPEs) can modify the strength of pest regulation, causing positive or negative deviations from those that are predicted from independent effects of isolated predators. Despite increasing evidence that omnivory can shape predator-prey interactions, few studies have examined the impact of alternative plant food on interactions between multiple predators. In the present study, we examined the effects and interactions of two omnivorous mirids, *Μacrolophus pygmaeus* and *Nesidiocoris tenuis*, on different densities of their aphid prey, *Myzus persicae*. Prey were offered to the to single or pairs of mirid predator individuals, either conspecific or heterospecific on a leaf, while simultaneously adding or excluding a flower as an alternative food resource. Data were compared with calculated expected values using the multiplicative risk model and the substitutive model. We showed that predation of aphids was reduced in the presence of the alternative flower resource in treatments with single *M*. *pygmaeus* individuals, but not with single *N*. *tenuis* individuals. When the predators had access only to prey, the effects of multiple predation, either conspecific or heterospecific, were additive. The addition of an alternative plant resource differently affected MPEs depending on the nature of predator pairings. Predation risk was increased in conspecific *M*. *pygmaeus* treatments at intermediate prey densities, whereas it was reduced in conspecific *N*. *tenuis* treatments at high prey densities. Observations of foraging behaviour concerning the location of conspecific pairings revealed that *M*. *pygmaeus* individuals showed a clear tendency to reside mainly in the flower, whereas *N*. *tenuis* individuals were found to reside at different posts in the dish. We suggest that the competition between omnivorous predators may be mediated through the diversity of their plant feeding preferences, which directly affects the strength of MPEs. Consequently, the preferences of the interacting predators for different plant resources should be considered in studies evaluating the outcomes of MPEs.

## Introduction

Intra- and interspecific interactions among organisms influence the complexity and resilience of natural and managed food webs (such as agricultural systems). In particular, there has been a rapid growth of interest in the regulation of prey populations emerging from the presence of multiple natural enemies [1, 2]. Emergent effects of predator assemblages occur when the number of prey consumed in the presence of multiple predator species differs from the summed effects of each predator in isolation [1–7]. Multiple-predator effects (MPEs) can be non-additive through either ‘risk enhancement’, in which prey survival in the presence of two predators is reduced as a result of diet complementarity or facilitation between predators, or ‘risk reduction’, in which prey survival in the presence of both predators is higher than predicted from their individual impacts in each single predator treatment (e.g., as a consequence of intraguild predation or omnivory) [3]. A neutral impact of multiple predators on the shared prey may also emerge when the effect of a predator species is not influenced by the presence of other predator species [6, 8].

A growing number of studies demonstrate that MPEs can be altered by the foraging mode [9], prey density [10], habitat complexity [11] or the presence of non-native prey [12]. Furthermore, it is well documented that plant food resources may substantially influence the effects of omnivorous predators on herbivore populations [13–19]. Numerous studies have confirmed that plant-provided food may result in reduced prey consumption by omnivorous predators if these plant resources and prey are substitutable [14, 20, 21]. According to Sabelis & van Rijn [20], the presence of plant resources can increase or decrease predation depending on the degree to which these resources are complementary or substitutable to the prey. However, all these studies have focused only on a single prey and single predator species.

Nevertheless, our knowledge of the effect of alternative food on MPEs is very limited. Venzon *et al*. [22], stated that the sign and the strength of MPEs on pests may change with the occurrence of omnivory in the food web. However, intraspecific competition for plant resources may increase prey consumption rates and enhance prey suppression. Interspecific competition, on the other hand, may also lead to increased prey consumption rates [23]. Thus, assessing whether the rates of phytophagy are associated with the prey consumption rate in a system of various predator species with different plant food requirements may yield valuable information from a theoretical and practical point of view. However, the way in which alternative plant resources may alter MPEs is still a novelty. In a very recent study, Wilby *et al*. [24] showed strong effects of plant composition on emergent multi-predator effects under short temporal and spatial scales. They suggest that the provision of extra-floral nectar by one of the two plant species tested can modify the strength and sign of emergent MPEs on prey population regulation. According to their results, a positive emergent effect in a wheat monoculture was reversed to a negative multi-predator effect in a wheat and faba bean polyculture by the change in foraging behaviour of individual predators in the presence of extra-floral nectar produced by the bean. Hence, it may be hypothesized that MPEs vary between plants before and after flowering, because the predator species involved may have different preferences for the food resources (i.e., pollen, nectar) typically available in flowers [25].

*Μacrolophus pygmaeus* Rambur and *Nesidiocoris tenuis* Reuter (Hemiptera: Miridae) are polyphagous predators and are commonly used in pest management of whiteflies and lepidopteran species, [26–36]. *Macrolophus pygmaeus* can survive in the absence of prey by feeding on plant sap [28, 37], and pollen has been reported to favor its development and fecundity [38]. Although *M*. *pygmaeus* has not been seen predating other hemipteran predators [39], it interferes with parasitoid biocontrol agents [32, 40] and displays a cannibalistic behaviour [41] *Nesidiocoris tenuis* is also a plant feeding generalist predator but cannot reach adulthood on plants in the absence of prey [42], although this may depend on the plant [43]. However, this species has been reported to not only benefit tomato plants directly by entomophagy but also indirectly by phytophagy, which induces a physiological response in the tomato plant [44].

These two predator species are likely to interact through MPEs, as there is evidence of reciprocal impacts on their behaviour and development when present together. Effects, such as changes in their distribution pattern on the plant and increased mobility, particularly of *N*. *tenuis* when both predators coexist, have been observed [45]. Adult females of *N*. *tenuis* cause high mortality of *M*. *pygmaeus* nymphs in the absence of prey [46]. Lampropoulos *et al*. [47] reported comparable con- and heterospecific interactions between the two mirids with reduction in prey risk and enhancement in prey risk for the whitefly *Trialeurodes vaporariorum* at intermediate and high levels of prey density, respectively. Moreover, Casula & Nannini [48] highlighted different MPEs between the mirid predators *N*. *tenuis* and *Macrolophus melanotoma* Costa depending on the prey density: the control of whitefly populations of *T*. *vaporariorum* by *M*. *melanotoma* was less effective than *N*. *tenuis* at high prey density, and vice versa at low prey density [48]. Finally, recent experimental evidence suggests that the presence of floral resources reduced the plateau of the functional response of *Μ*. *pygmaeus* on aphids [49].

In the present study, we investigated MPEs from two species of mirid predators by exposing conspecific and heterospecific pairs to different prey densities with and without access to a flower. This experimental system is amenable to testing the effects of different food resources on the outcome of MPEs. We specifically addressed the following questions: (a) Do *Μ*. *pygmaeus* and *N*. *tenuis* show conspecific and heterospesific interactions when foraging in a prey patch? (b) Are the above interactions affected by the level of prey availability? (c) Does flower availability affect the conspecific and heterospesific interactions, if any?

## Materials and Methods

### Biological materials

To examine the impact of multiple omnivorous predators on shared prey, we used *Μ*. *pygmaeus* and *N*. *tenuis*, thereafter indexed as *Mp* and *Nt*, which have comparable predation characteristics and a similar body size [26–32, 34, 37, 38, 42, 43, 45, 47]. A better control of the experimental conditions makes the results more reliable and increases our comprehension of the mechanisms at stake. For this reason, we simplified the prey component of the system by using only young instars of a mobile prey, the aphid *Myzus persicae* Sulzer (Homoptera: Aphididae).

*Μacrolophus pygmaeus* and *N*. *tenuis* rearings were initiated from adults and nymphs collected from a tomato field in Co. Boeotia, central Greece. Insects were reared on potted eggplants, *Solanum melongena* (cv Bonica), and provided with sufficient quantities of *Ephestia kuehniella* Zeller (Lepidoptera: Pyralidae) eggs as a food supply. Eggs of the Mediterranean flour moth *E*. *kuehniella* were obtained from Koppert BV (Entofood, The Netherlands). Rearing of the aphids was established on eggplant. Cultures of plants and all insect species were maintained in wood-framed cages (length 80 cm x height 70 cm), in a greenhouse kept at 22.5 ± 2.5°C under natural lighting conditions.

### Experimental design

The experimental set-up consisted of Petri dishes (Ø 9 cm, 1.5 cm height) with a mesh-covered hole in the lid (Ø 3 cm) to reduce the accumulation of humidity. A leaf of eggplant was placed, abaxial surface up, on a layer of water-moistened cotton wool on the bottom of each Petri dish. In all of the experiments, the fifth instar nymphs of *Mp* and/or *Nt* that were used were less than 24 hr of age. These were obtained from the nymphs of 2^nd^ instar that were transferred from wood-framed rearing cages to cages with potted eggplants with eggs of *E*. *kuehniella* at 25°C, 65 ± 5% RH and 16 hr light per day, and left to develop until the 5^th^ instar. Then, the nymphs were introduced to caged eggplants and were deprived of prey for 24 hours prior to beginning the experiments to exclude the influence of variable hunger levels. As a supplementary food source for the experiments, full bloom flowers of eggplants that were free of prey and insecticides were collected every day.

Single and multiple predator treatments were conducted in growth chambers under controlled conditions of 25°C, 65 ± 5% RH and 16 hr light per day. In single predator treatments, a 5^th^ instar *Mp* or *Nt* nymph was introduced in a dish with an eggplant leaf on which 2^nd^ instar *M*. *persicae* nymphs had been gently placed at various densities. Conspecific and heterospecific interactions were tested by introducing two predators (i.e., 2*Mp*, 2*Nt*, *MpNt*) into a dish with the eggplant leaf and the prey. All predator treatments were repeated with the presence of a flower in full bloom in addition, to the eggplant leaf and the prey in the Petri dish. The flower petioles were covered with moistened cotton to maintain their turgor. In each treatment, the prey densities were 4, 12, 20, 24, 32 and 40 prey individuals per dish, and 10 replicates were performed at each prey density. The location (in the flower or on the leaf) of each individual predator from each treatment was recorded visually after a 24-h period in all of the experimental trials. For heterospecific treatments, the nature of each predator (either *Nt* or *Mp*) was also recorded (which one was found on the leaf, which one in the flower). Then, the predators were removed from the dishes and the number of aphids consumed was recorded.

The natural mortality of aphids in dishes due to the experimental manipulations was evaluated by carrying out five replicates of each prey density in dishes without predators. The natural mortality rates were found to be negligible: 0.0, 0.0, 0.8 ± 0.37, 0.4 ± 0.24, 1.0 ± 0.32, and 1.2 ± 0.20 on eggplant leaf alone, and 0.0, 0.0, 0.0, 0.6± 0.24, 0.8± 0.20 and 1.0±0.0 (Mean ± SE) on eggplant leaf with the supplementary flower at densities of 4, 12, 20, 24, 32 and 40 aphids per dish, respectively.

### Data analysis

Analysis of the predation data was performed by a 3-way ANOVA with the first factor being the predator treatment (*Mp*, *Nt*, 2*Mp*, 2*Nt*, *MpNt*), the second factor being the prey density with six levels, and the third factor being the presence or absence of a flower along with the prey. Because the assumptions of normality were violated, log-transformation of data was used. For testing normality, the Shapiro-Wilk [50] procedure was used. Means were compared by a Student test.

To test simultaneously for independence and interactions between predator species both the ‘multiplicative risk model’ (MRM) and the substitutive model are needed to evaluate the impacts of the species composition and the density of predators [51, 52]. The multiplicative model allows to test if the expected prey consumption for two-predator combinations can be calculated from their individual consumption rates [3, 51], and it accounts for a reduction in prey that is available for each predator due to the presence of the other predator [3, 53, 54]. The substitution design determines if the predators are functionally substitutable by comparing mean per capita effects of individual predators to mean per capita effects of multiple predators [51, 55].

First, we examined whether positive (facilitative prey consumption) or negative (interference, prey risk reduction) interactions between individual predators in the conspecific and heterospecific treatments occurred according to the multiplicative risk model. If two predators *A* and *B* have independent effects, then the total expected proportion of prey killed by both predators *P*_*AB*_ should be:

$$P_{AB}=P_{A}+P_{B}-P_{A}P_{B}$$

where *P*_*A*_ and *P*_*B*_ are the probabilities of an aphid being consumed by the predator *A* or *B* in isolation respectively, and *P*_*A*_ *P*_*B*_ accounts for the prey consumed by one predator that are no longer available to the other [54]. For a given initial prey density *N*, the expected combined predation rate is thus *N*. *P*_*AB*_ over a 24-h period of exposure.

To test for MPEs in multiple–predator treatments, we pooled the observed and expected predation rates in a single response variable, on which we performed a 3-way ANOVA [3, 47, 51, 55]. The three factors were the following: (a) the predator treatment with three levels (2*Mp*; 2*Nt*; *MpNt*), (b) the type of data (observed versus expected value), and (c) the presence or absence of a flower. We fit full models including main effects and interactions between factors. Data used in the analysis were log transformed.

The intensity of the con- vs. heterospecific interactions was further examined with the substitutive model, which tests whether changes in absolute predation in two predator species differ from what is predicted based on combining individuals of the same species:

$$E_{\left(MpNt\right)}=\sqrt{Mp_{\left(1,2\right)}\times Nt_{\left(1,2\right)}}$$

where *E_(MpNt)_* is the expected predation rate, *Mp*_*(1*,*2)*_ and *Nt*_*(1*,*2)*_ are the observed predation rates by pairs of *Mp* and *Nt*, respectively [47, 51, 53]. The response variable for each treatment was the pooled number of prey rates (observed and expected obtained by this model). The data were analyzed by a 2 way-ANOVA with the first factor being the type of data (observed versus expected value), and with prey density as the second factor. Comparisons of the means were performed using a Student test.

To clarify if each individual has a similar tendency to reside on either the leaf or in the flower, the location of each individual in the dish (either on the leaf or in the flower) for both monospecific (*Mp* or *Nt*) or conspecific and heterospecific (2*Mp*, 2*Nt*, *MpNt*) treatments were independently analyzed by a chi-square test for each prey density in each treatment. Because no significant differences were found between prey densities within each predator treatment, the data of each prey density were pooled. The percentage of individuals in monospecific treatments found in the flower vs. on the leaf for each predator species was analyzed by a one-way ANOVA after arcsine transformation of data. For treatments with pairs of predators, three modalities were possible for the location of both individuals: both individuals gathered in the flower, both individuals gathered on the leaf, or one individual in the flower and one individual on the leaf. For this latter modality, we did not separate cases where *Nt* was found in the flower and *Mp* on the leaf from cases where *Mp* was found in the flower and *Nt* on the leaf. We used this information as a complement to the analysis. Percentages of treatments recorded in the three modalities were analyzed by a one-way ANOVA with the three location modalities being a three-level factor. All analyses were conducted in SAS 10.0.0 [56].

## Results

### Effects of treatments on overall prey consumption

We first tested the impact of predator combination, presence/absence of a flower and prey density on overall prey consumption. The 3-way ANOVA showed that the interaction of the factors “predator”, “presence or absence of a flower” and “prey density” was significant (F_20,540_ = 1.78; P = 0.02; Table 1).

**Table 1:** Number (mean ± SE) of prey consumed in monospecific (*Mp* or *Nt*), conspecific (2*Mp* or 2*Nt*) and heterospecific (*MpNt*) treatments, at various prey densities of *M*. *persicae* nymphs with or without the presence of a flower. *Mp* denotes *M*. *pygmaeus* and *Nt* denotes *N*. *tenuis*. Different upper letters indicate significant differences among prey densities for each treatment separately. Different lower letters indicate significant differences among treatments for each density separately.

	*Mp*		2*Mp*		*Nt*		2*Nt*		*MpNt*	
Prey Density	Prey	Prey +Flower	Prey	Prey +Flower	Prey	Prey +Flower	Prey	Prey +Flower	Prey	Prey +Flower
**4**	3.8 ±0.13 Aa	3.4 ± 0.27 Aa	3.9 ±0.10 Aa	3.8 ± 0.13 Aa	3.7 ±0.15 Aa	3.3 ±0.21 Aa	3.8 ±0.13 Aa	3.6 ±0.16 Aa	3.9 ±0.10 Aa	3.7 ±0.15 Aa
**12**	9.0 ±0.88 Bd	7.5 ± 1.15 Be	11.8 ±0.13 Ba	10.1 ±0.38 Babcd	9.7 ±0.62 Bbcd	9.0 ±0.58 Bcd	11.6 ±0.22 Bab	10.6 ±0.62 Bab	11.8 ±0.13 Ba	11.0 ±0.42 Bab
**20**	17.1 ±0.40 Cab	10.3 ±0.97 Cc	19.0 ±0.49 Ca	17.9 ±0.48 Cab	17.8 ±0.68 Cab	15.4 ±0.71 Cb	19.1 ±0.38 Ca	17.7 ±0.50 Cab	18.8 ±0.51 Ca	18.2 ±0.77 Ca
**24**	17.9 ±0.35 Ccd	9.8 ± 0.83 Ce	22.5 ±0.56 Da	19.7 ±1.04 Cabc	19.0 ±0.91 Cbcd	16.6 ±1.31 Cd	21.8 ±0.89 Cab	20.1 ±1.09 CDabc	22.6 ±0.56 Da	22.2 ±0.44 Da
**32**	18.9 ±0.31 Cd	11.9 ± 0.8 De	28.1 ±1.20 Ea	26.9 ±1.35 Dab	20.1 ±1.42 Ccd	19.5 ±1.36 Dd	27.5 ±0.79 Da	23.8 ±2.20 DEbc	28.5 ±1.30 Ea	25.8 ±0.85 Eab
**40**	22.8 ±0.49 Dd	15.1 ±0.62 Ee	33.7 ±0.92 Fa	28.0 ±2.70 Dbc	23.5 ±1.31 Dcd	26.1 ±1.59 Ecd	34.5 ±1.30 Ea	26.3 ±1.65 Ebc	33.5 ±1.65 Fa	30.4 ±1.16 Fab

In treatments without the provisioning of a flower, the prey consumption of a single individual of each species (*Mp* or *Nt*) did not differ between the two species (F_1,540_ = 1.29, P = 0.26; Table 1). Total prey consumption was significantly lower in single-predator treatments in comparison to both treatments with their respective conspecific pairs of predators (*Mp* vs. 2*Mp*: F _1,540_ = 52.37, P <0.0001; *Nt* vs. 2*Nt*: F _1,540_ = 33.25, P <0.0001) and to treatments with heterospecific pairs (*Mp* vs. *MpNt*: F _1,540_ = 51.69, P <0.0001; *Nt* vs. *MpNt*: F _1,540_ = 36.67, P <0.0001) (Table 1).

The provisioning of a flower in single *Mp* predator treatments resulted in a significant reduction of prey consumption at prey densities higher than 4 prey items (F _1,540_ = 141.58, P <0.0001), whereas in *Nt* treatments differences in predation were not observed (F _1,540_ = 4.01, P = 0.05). When conspecific pairs of each of the two predator species were used, the presence of a flower resulted in a significant decrease of the total prey consumption (F _1,540_ = 10.80, P = 0.001; F _1,540_ = 15.47, P <0.001 for 2*Mp* and 2*Nt*, respectively). Strikingly, the addition of a flower did not cause a significant effect in prey mortality caused by heterospecific pairs (*MpNt*) (F _1,540_ = 3.26, P = 0.07; Table 1).

### Multiple predator effects on prey consumption

**Fig 1:**
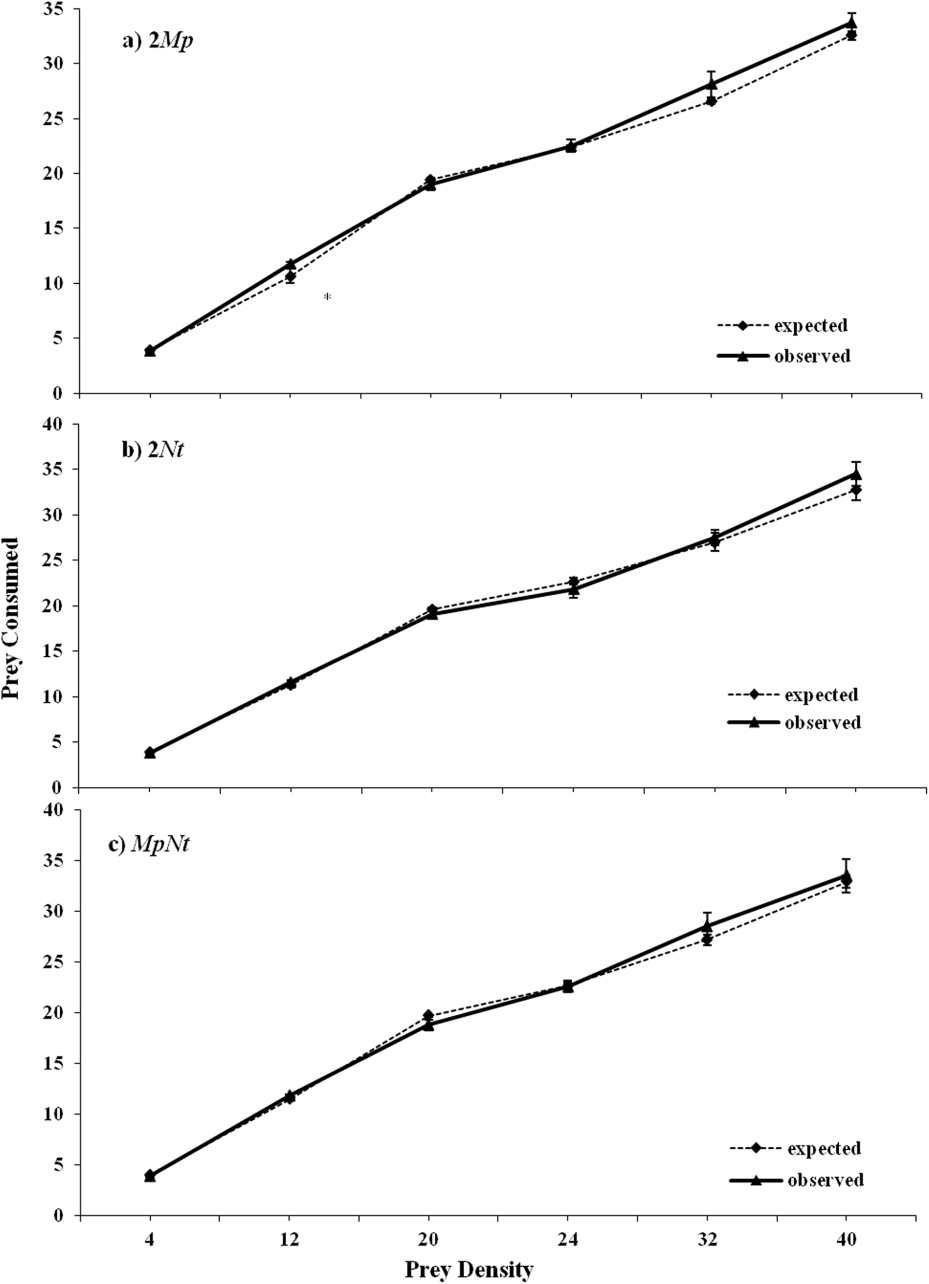
Observed prey consumption (mean ± SE) and prey consumption predicted by the multiplicative model for conspecific (2 *Mp* or 2*Nt*) or heterospecific (*MpNt*) pairings foraging on different densities of *M*. *persicae* nymphs. *Mp* denotes *M*. *pygmaeus* and *Nt* denotes *N*. *tenuis*. Asterisks indicate significant differences between the observed and predicted values of consumption (*P<0*.*05*).

We then studied the multiple predator effects on prey consumption by using multiplicative risk and substitutive models. When only prey were provided to predators, the observed proportions of prey eaten when two conspecifics or heterospecifics (2*Mp*, 2*Nt*, *MpNt*) foraged did not significantly differ from the expectations based on the multiplicative risk model, indicating that the predator effects were independent (Table 2, Fig 1).

**Table 2:** Results of ANOVAs used to compare observed predation by conspecific (2*Mp* or 2*Nt*) and heterospecific (*MpNt*) pairings to expected predation based on a multiplicative and substitutive experimental design. *Mp* denotes *M*. *pygmaeus* and *Nt* denotes *N*. *tenuis*.

	Prey				Prey & Flower			
**Source**	**df**	**SS**	**F**	**P**	**df**	**SS**	**F**	**P**
**Test of multiplicative design**								
Observed vs. expected	1	0.0011	0.7493	0.3874	1	0.0139	2.7483	0.0983
Predator (2*Mp*, 2*Nt*, *MpNt*)	2	0.0018	0.5929	0.5533	2	0.1872	18.5392	<0.0001
Observed vs. expected × Predator	2	0.0025	0.8415	0.4320	2	0.2037	20.1803	<0.0001
Density	5	28.5611	3866.579	<0.0001	5	25.4847	1009.791	<0.0001
Observed vs. expected × Density	5	0.0174	2.3614	0.0399	5	0.0631	2.4995	0.0307
Predator × Density	10	0.0048	0.3256	0.9741	10	0.0583	1.1552	0.3206
Observed vs. expected × Predator × Density	10	0.0049	0.3335	0.9717	10	0.0547	1.0830	0.3747
Error	324	0.4787				1.6354		
**Source**	**df**		**F**	**P**	**df**		**F**	**P**
**Test of substitutive design**								
Observed vs. expected	1	0.0001	0.0558	0.8138	1	0.0216	7.3433	0.0078
Prey density	5	9.6268	1188.163	<0.0001	5	8.8465	601.443	<0.0001
Observed vs. expected × Prey density	5	0.0016	0.2019	0.9610	5	0.0135	0.9142	0.4747
Error	108	0.1750			108	0.3177		

**Fig 2:**
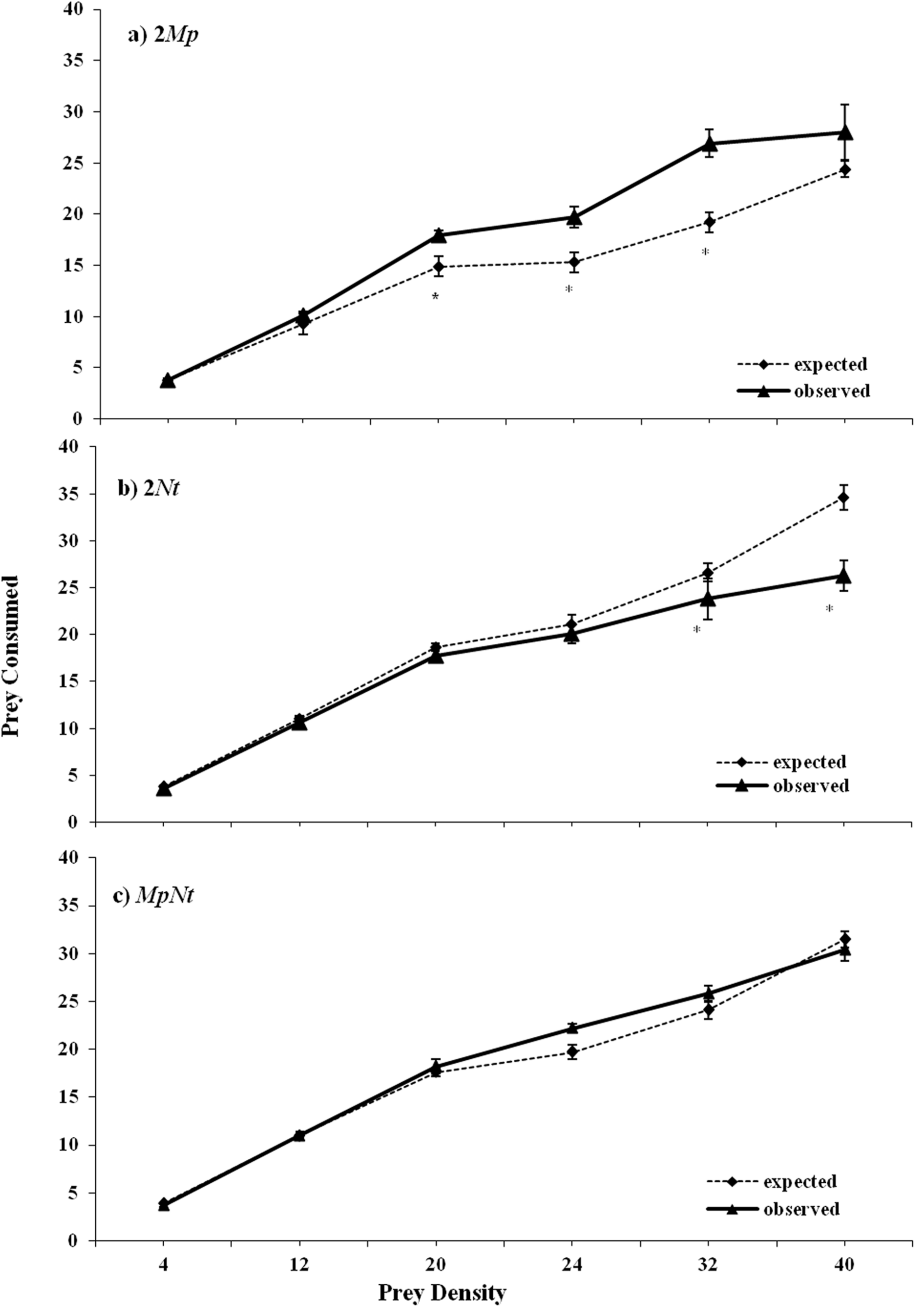
Observed prey consumption (mean ± SE) and prey consumption predicted by the multiplicative model for conspecific (2 *Mp* or 2*Nt*) or heterospecific (*MpNt*) pairings foraging on different densities of *M*. *persicae* nymphs and with the presence of a flower. *Mp* denotes *M*. *pygmaeus* and *Nt* denotes *N*. *tenuis*. Asterisks indicate significant differences between the observed and predicted values of consumption (*P<0*.*05*).

However, substantially different results were obtained when a flower was also provided to predators (Table 2, Fig 2). The multiplicative risk model revealed a risk-enhancing effect for prey in *Mp* conspecific treatments at the prey densities of 20, 24 and 32 individuals, whereas a risk-reducing effect was recorded in *Nt* conspecific treatments at the prey densities of 32 and 40 individuals (Fig 2A and 2B). However, in heterospecific treatments (*MpNt*), there was no evidence of an emergent MPE (Fig 2C).

**Fig 3:**
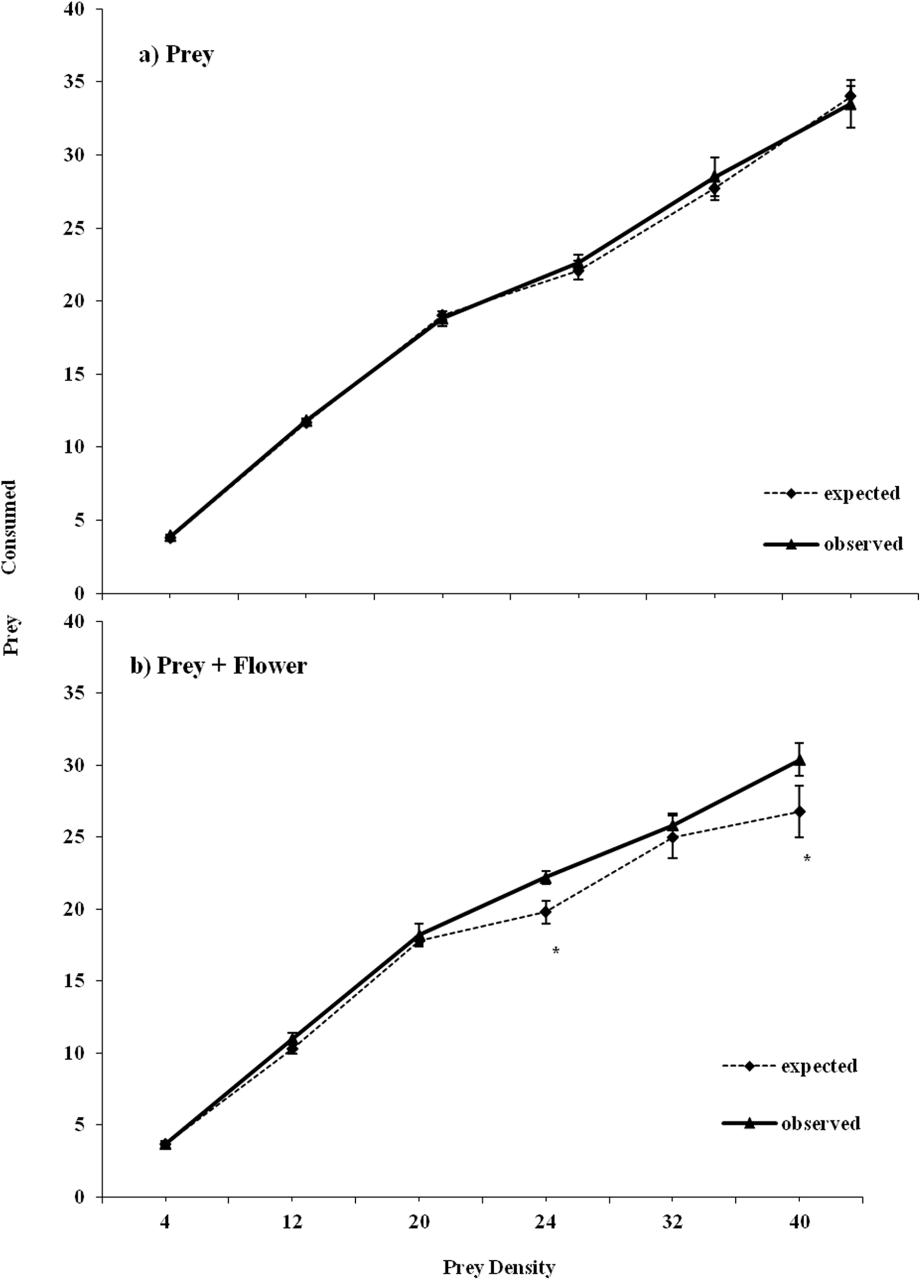
Observed prey consumption (mean ± SE) and prey consumption predicted by the substitutive model for heterospecific (*MpNt*) pairings foraging on different densities of *M*. *persicae* nymphs and with (a) and without (b) the presence of a flower. *Mp* denotes *M*. *pygmaeus* and *Nt* denotes *N*. *tenuis*. Asterisks indicate significant differences between the observed and predicted values of consumption (*P<0*.*05*).

The substitutive approach revealed no difference between the observed and expected prey consumption rates in the absence of a flower (Table 2; Fig 3A); by contrast, an emergent MPE was apparent on the mortality of their shared prey when a flower was available. The prey mortality was higher than the sum of the individual consumption of each species observed in the conspecific treatment and the results depended on prey density (Table 2, Fig 3B).

### Location of predator individuals: impact of predator species and of interactions between individuals

**Fig 4:**
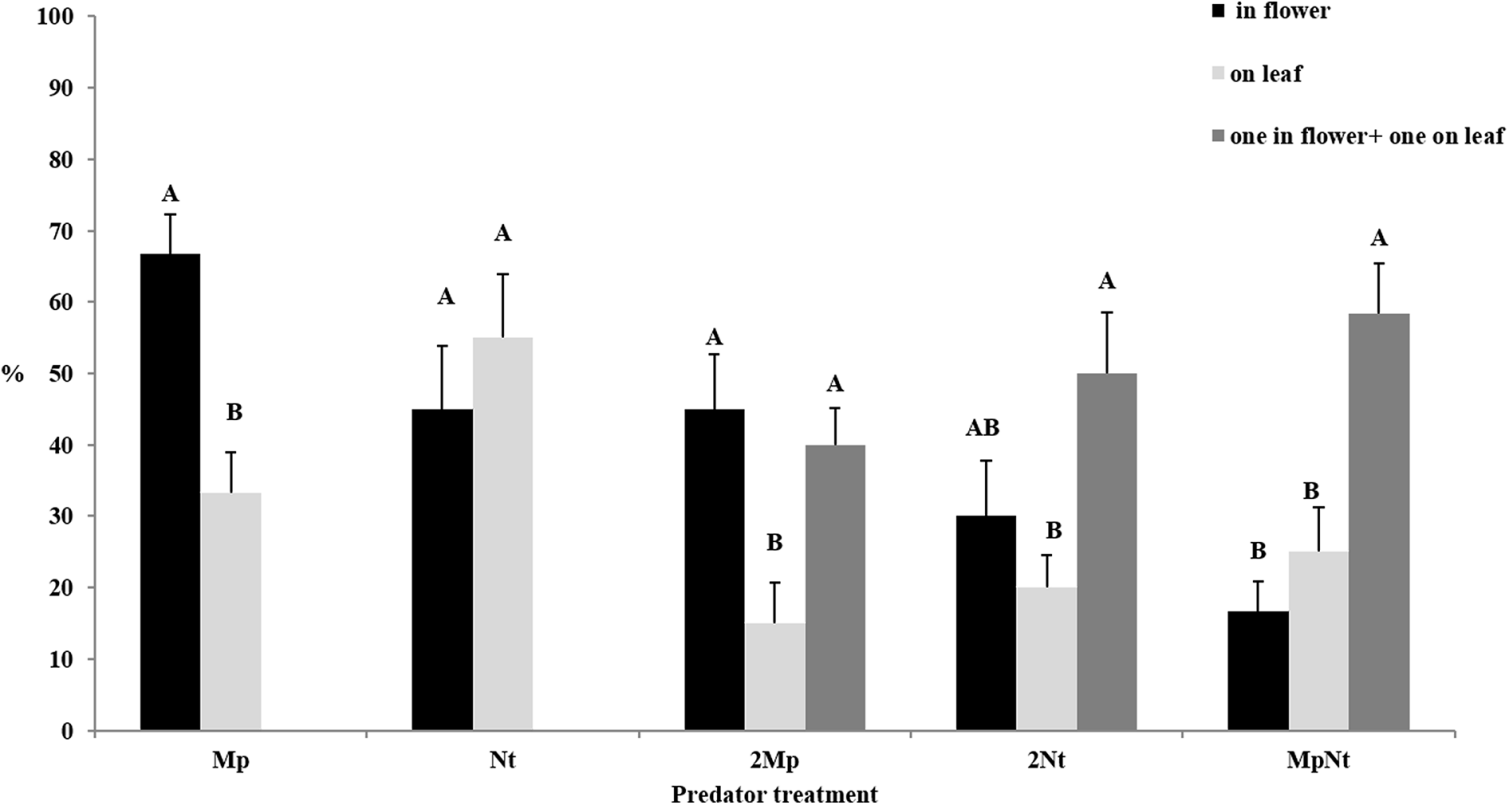
Percentages (%) of individuals in monospecific treatments (*Mp* or *Nt*) found in the flower or on the leaf and percentages of con- (2*Mp* or 2*Nt*) and heterospecific (*MpNt*) pairing treatments, where both individuals were found in the flower, on the leaf or one in the flower and one on the leaf. *Mp* denotes *M*. *pygmaeus* and *Nt* denotes *N*. *tenuis*. Same either lower or uppercase letters indicate significant differences in each predator treatment.

Finally, we studied the impacts of predator species (*Mp* vs. *Nt*) and interactions between individual predators on their location in the Petri dish (in the flower vs. on the leaf; Table 3; Fig 4).

**Table 3:** Number of individuals in monospecific (*Mp* or *Nt*) treatments found in the flower or on the leaf, and number of conspecific (2*Mp* or 2*Nt*) and heterospecific (*MpNt*) pairing treatments, where both individuals were found either in the flower or on the leaf, or were spatially separated when a leaf and a flower were present in the Petri-dish.

Prey density	*Mp*		*Nt*		2*Mp*			2*Nt*			*MpNt*		
	In Flower	On Leaf	In Flower	On Leaf	Both in Flower	Both on Leaf	1 Flower + 1 Leaf	Both in Flower	Both on Leaf	1 Flower +1 Leaf	Both in Flower	Both on Leaf	1 Flower +1 Leaf
**4**	9	1	6	4	6	1	3	6	1	3	1	1	8
**12**	7	3	7	3	6	1	3	1	1	8	2	2	6
**20**	6	4	5	5	5	2	3	4	2	4	2	3	5
**24**	7	3	5	5	4	0	6	1	2	7	0	3	7
**32**	5	5	3	7	5	1	4	3	4	3	3	1	6
**40**	6	4	1	9	1	4	5	3	2	5	2	5	3
**Sum**	40	20	27	33	27	9	24	18	12	30	10	15	35

The percentages of individuals in monospecific treatments that were found in the flower or on the leaf, and the percentages of pairings in con- and heterospecific treatments where both individuals were found in the flower, on the leaf or one in the flower and one on the leaf are presented in Fig 4. In the *Mp* monospecific treatment, a significantly higher percentage of individuals was recorded in the flower than on the leaf (F_1,10_ = 15.24, P = 0.0029), whereas no significant difference was recorded in the *Nt* treatment (F_1,10_ = 0.76, P = 0.4030). Individuals in *Mp* conspecific treatments were more often gathered in the flower or spatially separated than gathered on the leaf (F_2,15_ = 6.88, P = 0.0076) (Fig 4). In *Nt* conspecific treatments, most individuals were spatially separated, and the proportions of individuals gathered in the flower or on the leaf were equivalent (F_2,15_ = 4.64, P = 0.0270). In heterospecific treatments, the results were similar to *Nt* conspecific treatments, except that the proportion of individuals spatially separated was increased (F_2,15_ = 12.50, P = 0.0006) (Fig 4). In cases of heterospecific treatments where individuals were spatially separated, most of the individuals that were found in the flower were *Mp* (77.14%).

## Discussion

In the present study, we were interested in measuring multiple predator effects (MPEs) on prey consumption by two mirid bugs commonly used as biocontrol agents, *M*. *pygmaeus* and *N*. *tenuis*, and how these MPEs could be affected by the presence of an alternative flower resource for predators. Our lab experiment showed that both predators used the alternative flower resource differently. The presence of a flower lead to an enhanced predation risk in *Mp* conspecific treatments, whereas the predation risk was reduced in *Nt* conspecific treatments, and there was no significant MPE in treatments with heterospecific treatments. Finally, we found that *M*. *pygmaeus* individuals were more often in the flower than *N*. *tenuis* individuals, either alone or in pairs of both conspecific and heterospecific predators.

The provisioning of a flower to an individual *M*. *pygmaeus* yielded a significant reduction in the consumption of aphids, suggesting that this omnivorous species exploited these resources accordingly to their availability and that plant tissue and aphids are at least partially substitutable [57]. However, this reduction was not apparent in the treatments with a *N*. *tenuis* individual. Unlike *N*. *tenuis*, *M*. *pygmaeus* is able to develop while feeding exclusively on plant food without supplemental prey [28, 36, 37, 42, 43]. These differences in feeding ability of both predator species on plant versus animal resources may explain the observed differences in prey consumption in the single predator treatments.

Emergent MPEs by the multiplicative risk model did not occur in conspecific and heterospecific treatments when the predators had access only to prey. This suggests that the foraging effort and hunting behaviour of the predators were not strongly affected by the presence of another conspecific or heterospecific predator. The outcomes of the substitutive approach indicated also that additive effects of predation in two-predator systems compared with the one-predator systems hint at the absence of intra-guild predation and interference between predators, and this does not depend on the species of the predators. However, in another study [47], the two predator species used in our study produced emergent MPEs on the survival of *T*. *vaporariorum*, mainly at intermediate and high prey densities. This difference may be due to the mobile prey used in the present study influencing the outcome. Because these previous experiments took place on leaf disks, the increasing overlap of the search area by the predators might increase encounters with immobile prey to a greater extent than with mobile prey. Variation in predator impacts on prey can often be explained by variation in prey traits [58].

In the presence of the flower, the multiplicative risk design revealed that a risk-enhancing effect for prey occurred in *Mp* conspecific treatments, indicating the facilitation of the overall prey capture efficiency for conspecifics of *M*. *pygmaeus*. Conversely, a risk-reduction for prey occurred in *Nt* conspecific treatments, giving evidence for an emergent antagonistic effect for conspecifics of *N*. *tenuis*. In both treatments, the strength of the effects increased with prey density, indicating that the effect is driven by the low prey availability required by the predator to reach saturation [49]. The different effect that the flower elicited in conspecifics of *N*. *tenuis* may be the result of a potential interference between the predators, resulting in an emergent antagonistic effect and a decrease in prey consumption. Although no significant effect was found in the heterospecific treatment (*MpNt*) in the presence of a flower with the multiplicative risk approach, prey mortality was greater than predicted by the substitutive design. In many studies where the two approaches are used, different results have been obtained [52, 55, 59]. In the present study, conspecific interaction was found to be more intense than heterospecific interaction. Although it has been reported that con- and heterospecific interactions might be similar among closely related species [60], it seems that the two heteropteran species studied here may have foraging habits that are sufficiently different to minimize any negative antagonistic heterospecific interactions. This may be due to the differential dietary preferences and requirements of each predator species as argued in previous studies [26, 28, 37].

Such an interpretation of our results appears to be supported by the results of the foraging observations concerning the location of individuals either in the flower or on the leaf. In particular, single *M*. *pygmaeus* individuals showed a clear preference to reside in the flower, and this led to lower consumption of prey on the leaf. Conversely, in *Mp* conspecific treatments, individuals showed a tendency to exploit flower resources as well as prey on the leaf. This led to less competitive interference and the higher consumption of prey. Unlike *M*. *pygmaeus*, *N*. *tenuis* individuals were found in equal proportions on the leaf and in the flower in single-predator treatments. However, in *Nt* conspecific treatments both individuals were mostly found spatially separated, which may have caused the observed prey consumption to be lower than predicted. This behaviour may indicate the presence of negative interferences between *N*. *tenuis* individuals, which may possibly take place and be more aggressive than in *M*. *pygmaeus* individuals [46].

Although we did not observe intraguild predation, we found that, in heterospecific pairings, individual predators exhibited a strong tendency towards foraging at different locations. As revealed by the single-predator treatments (see above), *M*. *pygmaeus* individuals seem to benefit more from the flower resource than do *N*. *tenuis* individuals. This may explain why most of the *M*. *pygmaeus* individuals were found in the flower in heterospecific treatments, increasing access to the resource while minimizing spatial overlap with *N*. *tenuis* individuals.

Although there was no evidence for MPEs between the two omnivorous species, the addition of a flower resource caused the emergence of interactions. In fact, the significance of plant quality on intraguild interactions among omnivores has long been recognized [61]; however, competitive strength between them might also be mediated through the diversification of their feeding preferences for plant resources. Actually, competition may cause a shift in niche partitioning of plant resources (i.e., flower vs. leaf), which consequently affects the extraguild prey consumption. Thus, these behavioural responses can affect patterns of consumption and suggest a significant role of non-trophic interactions [62].

The role of predator diversity in maintaining ecosystem function and providing ecosystem services such as pest control are controversial [1, 2, 4, 63]. Therefore, knowledge of natural enemies’ abundance, composition, and complementarity is essential for a successful biological pest control program [25, 64]. Interestingly, the present results indicate that the effectiveness of omnivorous predators may be affected by the flowering of the crops, an understanding that may be useful in planning conservation or augmentative biological control programs. In conclusion, the preference of omnivorous predators for plant food resources (e.g., flowers) in association with the level of prey availability may substantially determine the outcome of con-or heterospecific interactions. However, our experiments considered only short-term interactions. Thus, there is a clear need for more long-term and larger spatial-scale studies involving more prey species to further explore the role of these interactions in population dynamics. This may produce important knowledge about resource use among and between omnivorous predators and will improve our ability to predict how predator effects and the presence of alternative food resources affect herbivore pest regulation.

## Supporting Information

S1 Table Raw data of prey consumed in monospecific (Mp or Nt), conspecific (2Mp or 2Nt) and heterospecific (MpNt) treatments at various prey densities of *M*. *persicae* nymphs with or without the presence of a flower. Mp denotes *M*. *pygmaeu*s and Nt denotes *N*. *tenuis*.
(PDF)

S2 Table Raw data of expected prey consumed in conspecific (2Mp or 2Nt) and heterospecific (MpNt) treatments at various prey densities of *M*. *persicae* nymphs with or without the presence of a flower according to MRM. Mp denotes *M*. *pygmaeus* and Nt denotes *N*. *tenuis*.
(PDF)

S3 Table Raw data of expected prey consumed in heterospecific (MpNt) treatments at various prey densities of *M*. *persicae* nymphs with or without the presence of a flower according to substitutive model. Mp denotes *M*. *pygmaeus* and Nt denotes *N*. *tenuis*.
(PDF)
