# Supplementary material for: Plant Resources as a Factor Altering Emergent Multi-Predator Effects
Source: PLoS One. 2015 Sep 25;10(9):e0138764. doi: 10.1371/journal.pone.0138764 (PMC4583265; doi:10.1371/journal.pone.0138764)
Supplement: S1 Table — Mp denotes M. pygmaeus and Nt denotes N. tenuis. (PDF) [file pone.0138764.s001.pdf]

S1 Table: Raw data of prey consumed in monospecific (Mp or Nt), conspecific (2Mp or 2Nt) and heterospecific (MpNt) treatments at various prey densities of M. persicae nymphs with or without the presence of a flower. Mp denotes M. pygmaeus and Nt denotes N. tenuis

	Only prey					Prey+Flower				
**Density**	**Mp**	**2Mp**	**Nt**	**2Nt**	**MpNT**	**Mp**	**2Mp**	**Nt**	**2Nt**	**MpNt**
**4**	4	4	4	3	3	4	4	2	3	4
**4**	4	4	4	4	4	4	4	4	4	3
**4**	4	4	4	4	4	4	4	4	4	4
**4**	4	4	3	3	4	4	4	4	3	4
**4**	4	4	4	4	4	2	3	3	4	4
**4**	3	4	4	4	4	3	4	3	4	4
**4**	4	4	3	4	4	2	4	4	4	3
**4**	3	3	3	4	4	4	4	3	3	3
**4**	4	4	4	4	4	**4**	3	3	3	4
**4**	4	4	4	4	4	**3**	4	3	4	4
**12**	6	12	9	12	12	9	9	8	12	8
**12**	8	12	12	12	12	10	9	11	6	12
**12**	3	12	11	12	12	11	10	11	12	11
**12**	12	12	11	12	11	12	10	5	11	12
**12**	10	12	11	12	12	11	9	8	12	11
**12**	12	11	6	10	12	6	12	8	10	10
**12**	9	12	7	12	12	5	12	10	10	12
**12**	10	12	9	11	11	6	10	10	9	12
**12**	11	11	11	12	12	1	11	9	12	10
**12**	9	12	10	11	12	4	9	10	12	12
**20**	18	15	20	20	18	4	18	15	17	20
**20**	15	20	18	20	18	9	19	17	19	20
**20**	17	20	16	20	20	9	18	14	19	17
**20**	20	19	15	20	20	10	20	17	15	13
**20**	16	20	18	20	18	15	18	19	19	19
**20**	17	19	14	18	20	14	19	16	18	19
**20**	18	18	20	18	15	12	15	17	18	20
**20**	15	20	18	17	20	11	19	14	15	15
**20**	17	20	19	18	20	10	16	14	18	20
**20**	18	19	20	20	19	9	17	11	19	19
**24**	19	24	15	20	24	7	20	18	18	22
**24**	17	20	18	23	18	8	18	21	18	22
**24**	19	22	22	23	23	6	20	18	13	24
**24**	18	24	21	24	23	8	23	19	24	23
**24**	18	19	18	15	22	10	19	18	18	24
**24**	19	24	24	20	22	12	22	13	23	20
**24**	19	22	18	24	23	12	12	11	24	20
**24**	17	24	15	23	24	9	23	9	22	22
**24**	17	23	20	22	24	14	22	21	20	23
**24**	16	23	19	24	23	12	18	18	21	22
**32**	20	25	15	30	27	16	25	17	31	23
**32**	20	20	22	24	29	14	32	19	26	27
**32**	19	31	15	24	29	16	31	24	10	30
**32**	17	28	29	31	32	12	26	28	22	29
**32**	18	32	24	28	32	10	26	15	27	27

**32**	19	30	22	25	27	10	30	23	32	24
**32**	19	32	20	30	18	10	17	20	18	24
**32**	20	25	15	28	31	11	28	15	31	23
**32**	19	29	19	27	31	10	29	16	20	23
**32**	18	29	20	28	29	10	25	18	21	28
**40**	22	38	15	38	30	17	16	30	33	33
**40**	22	30	27	40	37	17	20	25	20	36
**40**	23	35	20	30	32	15	37	30	33	30
**40**	24	35	26	31	22	13	27	29	20	25
**40**	22	30	25	29	30	13	36	24	22	27
**40**	25	34	24	36	36	14	39	28	30	35
**40**	20	33	21	40	40	17	19	17	22	32
**40**	22	37	30	31	35	18	22	18	24	30
**40**	23	35	24	34	38	14	36	31	29	26
**40**	25	30	23	36	35	13	28	29	30	30
