# Supplementary material for: Plant Resources as a Factor Altering Emergent Multi-Predator Effects
Source: PLoS One. 2015 Sep 25;10(9):e0138764. doi: 10.1371/journal.pone.0138764 (PMC4583265; doi:10.1371/journal.pone.0138764)
Supplement: S2 Table — Mp denotes M. pygmaeus and Nt denotes N. tenuis. (PDF) [file pone.0138764.s002.pdf]

S2 Table: Raw data of expected prey consumed in conspecific (2Mp or 2Nt) and heterospecific (MpNt) treatments at various prey densities of M. persicae nymphs with or without the presence of a flower according to MRM. Mp denotes M. pygmaeus and Nt denotes N. tenuis

		Prey			Prey+Flower	
**Density**	**2Mp**	**2Nt**	**MpNt**	**2Mp**	**2Nt**	**MpNt**
**4**	4	4	4	4	3	4
**4**	4	4	4	4	4	4
**4**	4	4	4	4	4	4
**4**	4	4	4	4	4	4
**4**	4	4	4	3	4	4
**4**	4	4	4	4	4	4
**4**	4	4	4	3	4	4
**4**	4	4	4	4	4	4
**4**	4	4	4	4	4	4
**4**	4	4	4	4	4	4
**12**	9	11	11	11	11	11
**12**	11	12	12	12	12	12
**12**	5	12	11	12	12	12
**12**	12	12	12	12	8	12
**12**	12	12	12	12	11	12
**12**	12	9	12	9	11	10
**12**	11	10	11	8	12	11
**12**	12	11	12	9	12	11
**12**	12	12	12	2	11	9
**12**	11	12	12	7	12	11
**20**	20	20	20	7	19	16
**20**	19	20	20	14	20	18
**20**	20	19	19	14	18	17
**20**	20	19	20	15	20	19
**20**	19	20	20	19	20	20
**20**	20	18	19	18	19	19
**20**	20	20	20	17	20	19
**20**	19	20	20	16	18	17
**20**	20	20	20	15	18	17
**20**	20	20	20	14	16	15
**24**	23	21	22	12	23	20
**24**	22	23	22	13	24	22
**24**	23	24	24	11	23	20
**24**	23	24	23	13	23	21
**24**	23	23	23	16	23	21
**24**	23	24	24	18	19	19
**24**	23	23	23	18	17	18
**24**	22	21	21	15	15	15
**24**	22	23	23	20	24	23
**24**	21	23	22	18	23	21
**32**	28	23	26	24	25	25
**32**	28	29	28	22	27	25
**32**	27	23	25	24	30	28
**32**	25	32	31	20	32	30

**32**	26	30	29	17	23	20
**32**	27	29	28	17	29	26
**32**	27	28	27	17	28	24
**32**	28	23	26	18	23	21
**32**	27	27	27	17	24	21
**32**	26	28	27	17	26	22
**40**	32	24	29	27	38	34
**40**	32	36	34	27	34	31
**40**	33	30	32	24	38	34
**40**	34	35	34	22	37	33
**40**	32	34	33	22	34	29
**40**	34	34	34	23	36	32
**40**	30	31	31	27	27	27
**40**	32	38	36	28	28	28
**40**	33	34	33	23	38	34
**40**	34	33	34	22	37	33
