# Supplementary material for: Plant Resources as a Factor Altering Emergent Multi-Predator Effects
Source: PLoS One. 2015 Sep 25;10(9):e0138764. doi: 10.1371/journal.pone.0138764 (PMC4583265; doi:10.1371/journal.pone.0138764)
Supplement: S3 Table — Mp denotes M. pygmaeus and Nt denotes N. tenuis. (PDF) [file pone.0138764.s003.pdf]

S3 Table: Raw data of expected prey consumed in heterospecific (MpNt) treatments at various prey densities of M. persicae nymphs with or without the presence of a flower according to substitutive model. Mp denotes M. pygmaeus and Nt denotes N. tenuis

	Prey	Prey+Flower
**Density**	**MpNt**	**MpNt**
**4**	3	3
**4**	4	4
**4**	4	4
**4**	3	3
**4**	4	3
**4**	4	4
**4**	4	4
**4**	3	3
**4**	4	3
**4**	4	4
**12**	12	10
**12**	12	7
**12**	12	11
**12**	12	10
**12**	12	10
**12**	10	11
**12**	12	11
**12**	11	9
**12**	11	11
**12**	11	10
**20**	17	17
**20**	20	19
**20**	20	18
**20**	19	17
**20**	20	18
**20**	18	18
**20**	18	16
**20**	18	17
**20**	19	17
**20**	19	18
**24**	22	19
**24**	21	18
**24**	22	16
**24**	24	23
**24**	17	18
**24**	22	22
**24**	23	17
**24**	23	22
**24**	22	21
**24**	23	19
**32**	27	28
**32**	22	29
**32**	27	18
**32**	29	24

**32**	30	26
**32**	27	31
**32**	31	17
**32**	26	29
**32**	28	24
**32**	28	23
**40**	38	23
**40**	35	20
**40**	32	35
**40**	33	23
**40**	29	28
**40**	35	34
**40**	36	20
**40**	34	23
**40**	34	32
**40**	33	29
